# Systemic EP4 Inhibition Increases Adhesion Formation in a Murine Model of Flexor Tendon Repair

**DOI:** 10.1371/journal.pone.0136351

**Published:** 2015-08-27

**Authors:** Michael B. Geary, Caitlin A. Orner, Fatima Bawany, Hani A. Awad, Warren C. Hammert, Regis J. O’Keefe, Alayna E. Loiselle

**Affiliations:** 1 Center for Musculoskeletal Research, University of Rochester, Rochester, New York, United States of America; 2 School of Medicine and Dentistry, University of Rochester, Rochester, New York, United States of America; 3 Department of Orthopaedics and Rehabilitation, University of Rochester, Rochester, New York, United States of America; 4 Department of Biomedical Engineering, University of Rochester, Rochester, New York, United States of America; University of Birmingham, UNITED KINGDOM

## Abstract

Flexor tendon injuries are a common clinical problem, and repairs are frequently complicated by post-operative adhesions forming between the tendon and surrounding soft tissue. Prostaglandin E2 and the EP4 receptor have been implicated in this process following tendon injury; thus, we hypothesized that inhibiting EP4 after tendon injury would attenuate adhesion formation. A model of flexor tendon laceration and repair was utilized in C57BL/6J female mice to evaluate the effects of EP4 inhibition on adhesion formation and matrix deposition during flexor tendon repair. Systemic EP4 antagonist or vehicle control was given by intraperitoneal injection during the late proliferative phase of healing, and outcomes were analyzed for range of motion, biomechanics, histology, and genetic changes. Repairs treated with an EP4 antagonist demonstrated significant decreases in range of motion with increased resistance to gliding within the first three weeks after injury, suggesting greater adhesion formation. Histologic analysis of the repair site revealed a more robust granulation zone in the EP4 antagonist treated repairs, with early polarization for type III collagen by picrosirius red staining, findings consistent with functional outcomes. RT-PCR analysis demonstrated accelerated peaks in *F4/80* and type III collagen (*Col3a1*) expression in the antagonist group, along with decreases in type I collagen (*Col1a1*). *Mmp9* expression was significantly increased after discontinuing the antagonist, consistent with its role in mediating adhesion formation. *Mmp2*, which contributes to repair site remodeling, increases steadily between 10 and 28 days post-repair in the EP4 antagonist group, consistent with the increased matrix and granulation zones requiring remodeling in these repairs. These findings suggest that systemic EP4 antagonism leads to increased adhesion formation and matrix deposition during flexor tendon healing. Counter to our hypothesis that EP4 antagonism would improve the healing phenotype, these results highlight the complex role of EP4 signaling during tendon repair.

## Introduction

Flexor tendons (FT) in the hand run on the palmar side of the digits and transmit the forces that allow for finger flexion. Primary repair of FT injuries in zone II remains a challenging surgical problem with a high rate of post-operative complications [1–4]. Fibrous adhesions between the tendon and surrounding tissue form to some extent in all cases of tendon repair, and up to 30–40% of cases are significant enough to result in loss of digit range of motion (ROM) and impaired hand function [5]. There are more than 30,000 tendon repair procedures a year in the US, with billions in associated healthcare costs [6]. Given this clinical challenge, there is significant interest in optimizing the repair process to improve functional outcomes following FT injury.

Great progress has been made through improving suture techniques and early rehabilitation protocols following FT surgery [7, 8], and while functional outcomes have benefited from such protocols, there remains room for improvement. One area of interest for improving FT repair is the fibrous adhesions that form as a result of excessive inflammation around the injury site [1, 9, 10]. While some degree of inflammation is necessary for repair, excessive extracellular matrix (ECM) deposition can disrupt the near-frictionless environment of the FT gliding within its synovial sheath [11, 12]. Thus, attenuating ECM deposition after injury is an apt target for improving outcomes after FT surgery.

Previous studies have targeted the inflammatory cascade in an effort to improve tendon healing [13–16]. Common among these studies has been the use of Cox-2 inhibitors, which have repeatedly shown concomitant losses in the strength of the repair, a concerning outcome for tissues that experience high loads such as the flexor tendon. While inflammation is required for repair, including recruitment of new cells that synthesize granulation tissue and collagen, excessive inflammation contributes to adhesion formation between the tendon and surrounding structures [1]. The challenge, therefore, becomes attenuating inflammation without weakening the biomechanics of the healing tendon.

Prostaglandin E2 (PGE2), an arachidonic acid metabolite, has been implicated as an inflammatory mediator in tendon injuries and tendinopathy [17–21]. PGE2 signals through one of four downstream receptors, EP1 through EP4, all of which belong to the super-family of G-protein coupled receptors [22]. Work by Thampatty *et al*., using human patellar tendon fibroblasts identified EP4 as the specific receptor mediating degenerative changes in tendinopathy [23], suggesting a potential therapeutic role for selective EP4 receptor antagonists. While significant work has been done investigating the fibrotic effects of modified PGE2-EP4 signaling in the lung [24, 25], and kidney [26, 27], its effects on tendon repair remain to be understood.

In an attempt to avoid the negative effects on mechanical properties seen in previous studies using anti-inflammatory treatment [13–16], we sought to modulate inflammation through inhibition of EP4, a downstream mediator of Cox-2/PGE2 inflammation. By targeting a specific receptor downstream of Cox-2/PGE2, only a subset of prostaglandin-mediated inflammation is inhibited, while non-EP4 mediated pathways are preserved. Further, EP4 antagonists do not have the same systemic side effects commonly associated with Cox-2 inhibitors [28], which is important to consider in any translational research.

In the present study we tested the hypothesis that inhibiting PGE2-EP4 signaling following flexor tendon injury will attenuate the inflammatory response and decrease adhesion formation without compromising the strength of the repair. To test this hypothesis, we utilized a murine model of flexor tendon laceration and repair in the hind-paw, and delivered a systemic EP4 antagonist during the late inflammatory and early proliferative phase of tendon healing. We analyzed the changes in digit flexion and gliding, the biomechanical strength of the repairs, the histologic changes within the area of repair, as well as changes in genes associated with tendon catabolism and repair.

## Materials and Methods

### Animal Ethics

This study was carried out in strict accordance with the recommendations in the Guide for the Care and Use of Laboratory Animals of the National Institutes of Health. All animal procedures were approved by the University Committee on Animal Research (UCAR) at the University of Rochester (UCAR Number: 2014–004). All surgery was performed in the morning in a designated small animal surgery room; animals were sedated using ketamine (60 mg/kg) and xylazine (4 mg/kg), and post-operative pain was managed with a single subcutaneous injection of 0.05mL extended-release buprenorphine (1.3 mg/mL). This protocol was based on previous studies from our group [29]. Up to five mice per cage were housed in a secure animal room with a 12 h light-dark cycle in cages with standard bedding. Animals were provided *ad lib* food and water, and any singly housed animals were provided small shacks for environmental enrichment. The animals’ health status was monitored throughout the experiments by a health surveillance program according to guidelines from the Association for Assessment and Accreditation of Laboratory Animal Care (AAALAC International). The mice were free of all viral, bacterial, and parasitic pathogens. Experimental animals were not used for breeding purposes.

### Murine Flexor Tendon Healing Model

Eight-to-ten week old female C57BL/6J mice (Jackson Laboratories, Bar Harbor, ME) underwent surgical transection and repair of the flexor digitorum longus (FDL) tendon as previously described (average weight 20 g, range 16–21g) [29, 30]. Briefly, the proximal FDL tendon was transected along the tibia at the myotendinous junction to protect the distal repair. The distal FDL tendon was exposed using a longitudinal incision along the plantar hind foot. The tendon was transected and then repaired using two horizontal 8–0 nylon sutures (Ethicon Inc., Summerville, NJ) in a modified Kessler pattern. The hind foot and tibial incisions were closed using a single 5–0 nylon suture (Ethicon Inc., Summerville, NJ). Post-operatively, mice were returned to their cage and allowed free active motion and weight bearing.

To suppress EP4 signaling, intraperitoneal injection of 10mg/kg EP4 antagonist (L161,982; Cayman Chemical Co, Ann Arbor, MI; CAS 147776-06-5) was administered on post-surgery days 5–8. Delayed EP4 antagonist treatment is based on previous studies demonstrating that delayed inhibition is preferable to immediate inhibition, since excessive inflammation and tissue remodeling are inhibited without disrupting the initial phases of healing [13]. Control groups were treated with the same weight-based doses of saline as a vehicle control. Mice were randomly assigned to treatment groups after surgery to avoid any surgeon-induced bias at the time of operation. Mice were sacrificed between post-operative days 3–28 for analysis of the outcomes described below.

### cAMP enzyme immunoassay (EIA)

At seven days post-surgery, repaired tendons were harvested from the distal aspect of the tarsal tunnel up until the tendon bifurcated into the digits (n = 3 per treatment group). On the day of sacrifice, mice were given their respective treatments in the morning, and then sacrificed 8 hours later. Each group therefore received a total of three treatments. cAMP EIA was performed according to the manufacturer’s protocol (Cayman Chemical Co, Ann Arbor, MI). Briefly, cAMP-acetylcholinesterase conjugate, mouse anti-cAMP monoclonal antibody, and either standard or sample was added to each well of pre-coated EIA plates. Standards and samples were both acetylated, and the samples were run in triplicates using 5- and 10-fold dilutions. Following 18h incubation at 25°C, the plate was washed and Ellman’s reagent was added to each well. Absorbance was determined at 405 mm and 420 mm by Synergy Mx Monochromator-based Microplate Reader (BioTek Instruments, Winooski, VT). Concentrations are expressed as picomoles per milliliter (pmol/mL).

### Adhesion Testing and Gliding Coefficient

Adhesion testing was performed at post-repair days 10, 14, 21, and 28 (n = 10–12 per treatment per time-point). Immediately following sacrifice, the hind limb was disarticulated at the knee, and the FDL tendon was released from the surrounding tissue proximal to the tarsal tunnel. The proximal end of the FDL tendon was secured between two pieces of tape. The limb was fixed in a custom apparatus with the tibia rigidly gripped to prevent rotation. To standardize the neutral position, the toes were passively extended by the examiner and allowed to return to an unloaded position before a digital image was taken to determine the neutral position (zero load) of the metatarsophalangeal (MTP) joint. Incremental loads were applied to the proximal end of the FDL and digital images were taken to quantify the MTP flexion angle relative to the neutral position. The MTP flexion angles were measured by two independent observers using ImageJ software (http://rsb.info.nih.gov/ij/), and plotted versus the applied load. The gliding resistance was determined by fitting the flexion data to a single-phase exponential equation where the MTP flexion angle = β x (1-exp(-m/α)); where m is the applied load (Prism GraphPad 6.0a; GraphPad Software, Inc., San Diego, CA). The curve fit was constrained to the maximum flexion angle (β) for normal tendons that was previously determined to be 75° for the 19g applied load [31]. Non-linear regression was used to determine the gliding resistance (α), which has been previously shown to correlate inversely with the range of MTP joint flexion [31]. Thus, the gliding resistance is a useful quantitative measure of the resistance to MTP flexion and correlates significantly with the work of joint flexion [32]. In addition, the MTP flexion range of motion (ROM) was determined as the difference in flexion angle between the applied loads of 0g and 19g.

### Biomechanical Testing

Following MTP flexion testing, the proximal extent of the FDL tendon was released from the tarsal tunnel, the calcaneus and tibia were removed, and the proximal end of the FDL was gripped in the Instron device (Instron 8841 DynaMight axial servohydraulic testing system, Instron Corporation, Norwood, MA), with the distal bones of the foot secured without disrupting the repair or branching tendon insertion into the phalanges. The tendon was tested in tension in displacement control at a rate of 30mm/minute until failure. Force-displacement data were automatically logged and plotted to determine the maximum load at failure (ultimate failure force) and tendon stiffness (slope of the linear portion of the load-deformation curve).

### RNA Extraction and Real-Time RT-PCR

Repaired tendons were harvested from the distal aspect of the tarsal tunnel up until the tendon bifurcated into the digits (n = 3 per treatment per time-point). Tendons from each time-point (3, 7, 10, 14, 21 days post-repair) were pooled and homogenized in Trizol reagent (Invitrogen, Carlsbad, CA) using the Ultra Turrax T8 homogenizer (IKA Works, Wilmington, NC). Five-hundred nanograms of RNA was reverse-transcribed to single-stranded cDNA using the iScript cDNA synthesis kit (BioRad, Hercules, CA). This cDNA served as a template for real-time PCR using PerfeCTa SYBR Green SuperMix (Quanta Biosciences, Gaithersburg, MD) and gene specific primers (Table 1). Gene expression was standardized to the internal control β-*actin* and normalized to day 3 expression levels. Neither group had received any treatment in the first three days post-repair, as such, day 3 repairs are not specific to either treatment group. Repaired tendons were harvested and analyzed in the same manner from a separate cohort of untreated eight-to-ten week old female C57BL/6J mice (Jackson Laboratories, Bar Harbor, ME) to characterize the temporal expression of *EP4* in our FT repair model.

**Table 1:** RT-PCR Primer Sequences.

Gene	Forward (5’–3’)	Reverse (5’–3’)
β-*Actin*	AGATGTGGATCAGCAAGCAG	GCGCAAGTTAGGTTTTGTCA
*F4/80*	CTTTGGCTATGGGCTTCCAGTC	GCAAGGAGGACAGAGTTTATCGTG
*Col3a1*	GCCCACAGCCTTCTACAC	CCAGGGTCACCATTTCTC
*Col1a1*	GAGCGGAGAGTACTGGATCG	GCTTCTTTTCCTTGGGGTTC
*Mmp9*	TGAATCAGCTGGCTTTTGTG	ACCTTCCAGTAGGGGCAACT
*Mmp2*	AGATCTTCTTCTTCAAAGGACCGGTT	GGCTCCTCAGTGGCTTGGGGTA

Forward and reverse primer sequences used for RT-PCR. Expression levels were normalized to the internal control β-actin, with each sample run in triplicates.

### Histology

Whole hind limbs containing the repaired tendons were harvested as previously described on post-repair days 10, 14, 21 and 28 (n = 4 per treatment per time-point) [30]. Briefly, the samples were fixed in 10% neutral buffered formalin for 48h with the tibia at 90° relative to the foot, then washed in PBS and decalcified in 14% EDTA (pH 7.2) for 14 days at room temperature. The decalcified tissues were put through a sucrose gradient, and embedded in OCT compound (Tissue-Tek, Sakura Finetek U.S.A., Inc., Torrance, CA). Serial eight-micron sagittal sections through the FDL tendon plane were then cut and stained with alcian blue/hematoxylin and Orange G or picrosirius red (Polysciences Inc., Warrington, PA). Picrosirius red sections were illuminated with monochromatic polarized light, providing a visualization of collagen fiber organization; collagen I appears red, while collagen III appears yellow/green [33, 34].

### Statistical Analysis

Results are shown as the mean ± standard error of the mean (SEM). Statistical significance was tested via multiple t-tests comparing antagonist and vehicle treated groups, correcting for multiple comparisons using the Holm-Sidak method with significance set at p<0.05. Group sizes were based on post-hoc power analysis of previous studies for biomechanical, gliding testing and qPCR analysis [30].

## Results

### EP4 Expression Peaked Early After Repair

**Fig 1:**
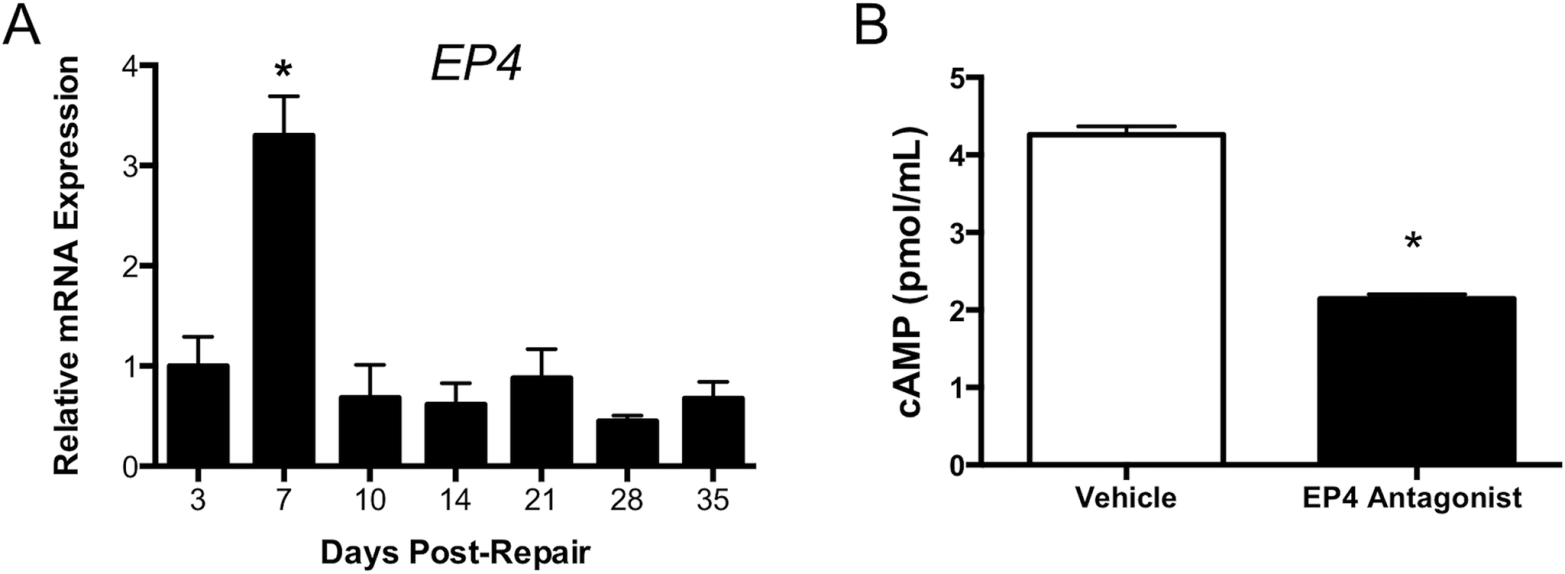
Temporal Expression of *EP4* and Effective Inhibition with Systemic EP4 Antagonism. **1A**: qPCR analysis of *EP4* expression in wilt-type tendons harvested between 3 and 28 days post-repair demonstrated a significant 2.3-fold increase in *EP4* at 7 days post-repair. **1B**: Local levels of cAMP were measured in repairs of EP4 antagonist (black bars) and vehicle treated (white bars) repairs 7 days after surgery. There were significant decreases in cAMP in the antagonist group, suggesting that the systemic EP4 antagonist effectively decreases EP4 signaling. *p<0.05.

An intrasynovial FDL tendon repair model was used in untreated mice to determine the expression profile of *EP4* (*Ptger4*), which would inform the timing for EP4 antagonist treatment in the study groups (Fig 1A). While prior studies have reported increases in *EP4* following tendon injury,[20, 21] it was important to characterize the temporal expression of *EP4* in our flexor tendon injury model in order to appropriately target the peak timing of *EP4* during the repair process. Expression peaked 7 days post-repair relative to day 3 (Day 3: 1.0 ± 0.3; Day 7: 3.3 ± 0.4,p<0.05); levels of *EP4* expression had returned to day 3 levels by 10 days post-repair. This significant and transient peak in *EP4* provided the rationale for treatment with the EP4 antagonist on days 5–8 post-repair.

### EP4 Signaling was Effectively Decreased by Systemic EP4 Antagonism

EP4 has been shown to exert its biological activity by increasing intracellular levels of cAMP [35]. As such, quantifying cAMP levels in the repair site from EP4 antagonist and vehicle treated mice would indicate whether EP4 signaling was effectively inhibited in the antagonist group. On day 7 post-repair the antagonist group had significant decreases in cAMP levels (Vehicle: 4.26 pmol/ml ± 0.11; Antagonist: 2.15 pmol/ml ± 0.06, p<0.05) (Fig 1B).

### EP4 Antagonism Decreased Range of Motion and Increased Gliding Resistance after Flexor Tendon Repair

**Fig 2:**
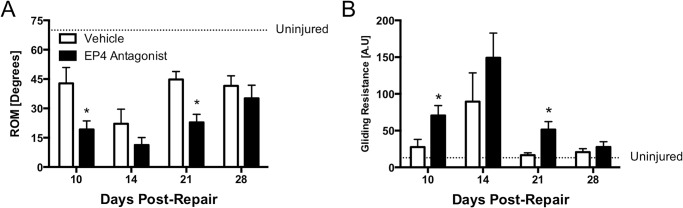
Systemic EP4 Antagonism Decreased Range of Motion and Increased Gliding Resistance after Flexor Tendon Repair. **2A**: Metatarsophalangeal joint range-of-motion was measured as the change in flexion angle from the unloaded to maximally loaded flexor digitorum longus tendon. Antagonist treated repairs (black bars) had significant decreases in range of motion at 10 and 21 days post-repair, suggesting increasing adhesion formation in the earlier time points. The differences between treatment groups were no longer present at 28 days post-repair. **2B**: Gliding resistance represents the overall work in bringing the hindpaw from the neutral to maximally flexed position, and is calculated from the force-flexion curve over incremental loading of the tendon. The resistance was significantly increased at 10 and 21 days post-repair in the antagonist treated group (black bars), suggesting greater adhesions creating resistance to digit flexion. *p<0.05.

Significant decreases in MTP ROM were seen at 10 days post-repair in the EP4 antagonist group (19.1° ± 4.4°) compared to vehicle treatment (42.8° ± 8.1°, p<0.05) (Fig 2A). This trend was maintained at 14 days, and ROM remained significantly lower in the antagonist group as far out as 21 days post-repair (Vehicle: 44.8° ± 4.1°; Antagonist: 22.9° ± 4.1°, p<0.05). There were no longer any significant differences in ROM between treatment groups at 28 days post-repair.

At 10 days post-repair, EP4 antagonist treated repairs had significantly higher gliding resistance than vehicle treated controls (Vehicle: 27.5 ± 10.6; Antagonist: 70.6 ± 12.8, p<0.05), consistent with the MTP ROM at the same time-point (Fig 2B). This difference was also seen at 21 days post repair (Vehicle: 44.8 ± 4.1; Antagonist: 22.9 ± 4.1, p<0.05). By day 28, there was no significant difference in gliding resistance between vehicle and antagonist treated repairs.

### Strength and Stiffness were Unchanged in Flexor Tendon Repairs of EP4 Antagonist Treated Repairs

**Fig 3:**
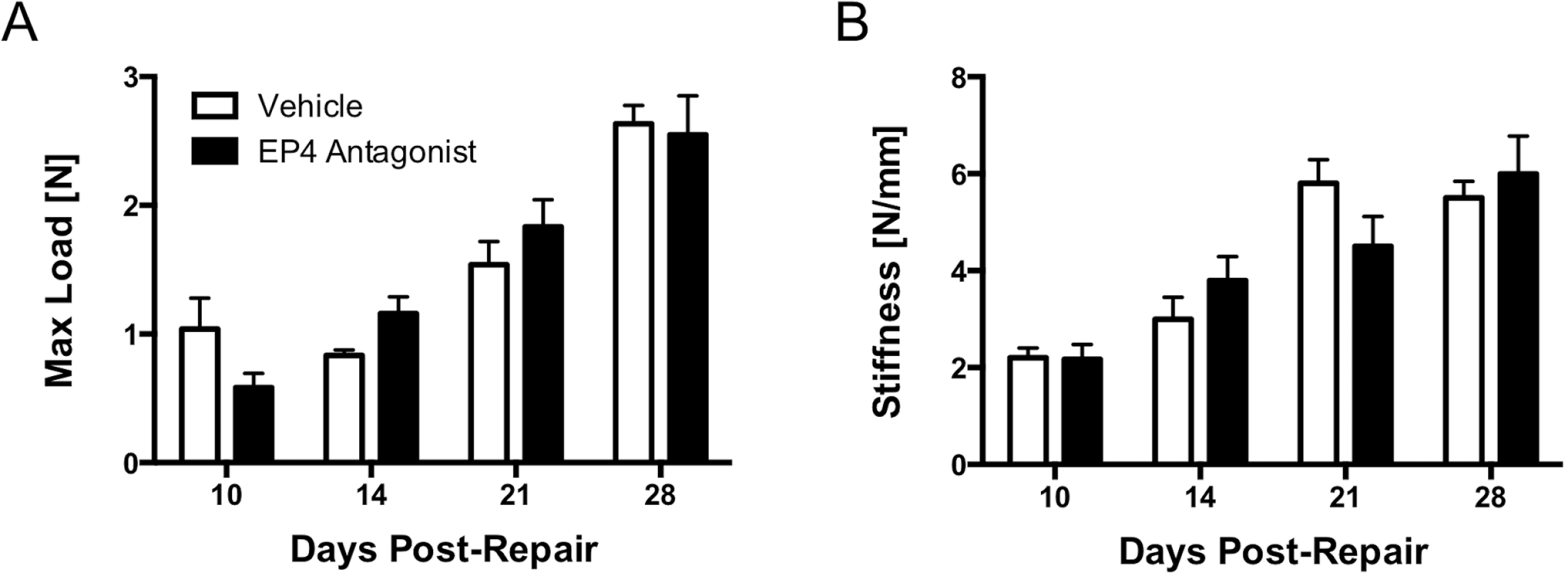
Maximum Load at Failure and Stiffness were Unchanged in EP4 Antagonist Treated Repairs. **3A**: Maximum load at failure, a measure of strength, was determined by tensile testing of the repaired flexor tendons. No significant differences were seen between EP4 antagonist (black bars) and vehicle treated repairs (white bars), with both groups exhibiting gradual increases in strength between 10 and 28 days. **3B**: Stiffness, the linear portion of the force-displacement curve generated during tensile testing, was no different between EP4 antagonist (black bars) and vehicle treated (white bars) repairs at any time-point. *p<0.05.

The maximum tensile load at failure of repaired tendons was determined in the EP4 antagonist and vehicle treated groups to evaluate changes in biomechanical properties of the repair. Both groups exhibit an expected decrease in the maximum load at failure early after repair, with gradual increases in the maximum load between 10 and 28 days post-repair (Fig 3A). Maximum load at failure in the vehicle treated group increased from 10 (1.1N ± 0.2N) to 28 days (2.6N ± 0.1N), while the EP4 antagonist group saw a similar increase over the same time (Day 10: 0.6N ± 0.1N; Day 28: 2.6N ± 0.3N). In the EP4 antagonist repairs, this represented a 3.4-fold increase in the maximum load at failure, suggesting that increasing strength is conferred over time. There were no significant differences between the two groups at any time-points. Similar to strength measurements, there is an initial decrease in stiffness early after repair (Day 10; Vehicle: 2.2 N/mm ± 0.2 N/mm; Antagonist: 2.2 N/mm ± 0.3 N/mm), with gradual increases through 28 days post-repair (Fig 3B). The stiffness of the repairs in the EP4 antagonist and vehicle treated groups were not significantly different at any time-points. No changes in mechanical properties were observed in un-injured contralateral control tendons from vehicle or EP4 antagonist treated mice.

### EP4 Antagonism Increased Early Granulation and Matrix Deposition around the Repair

**Fig 4:**
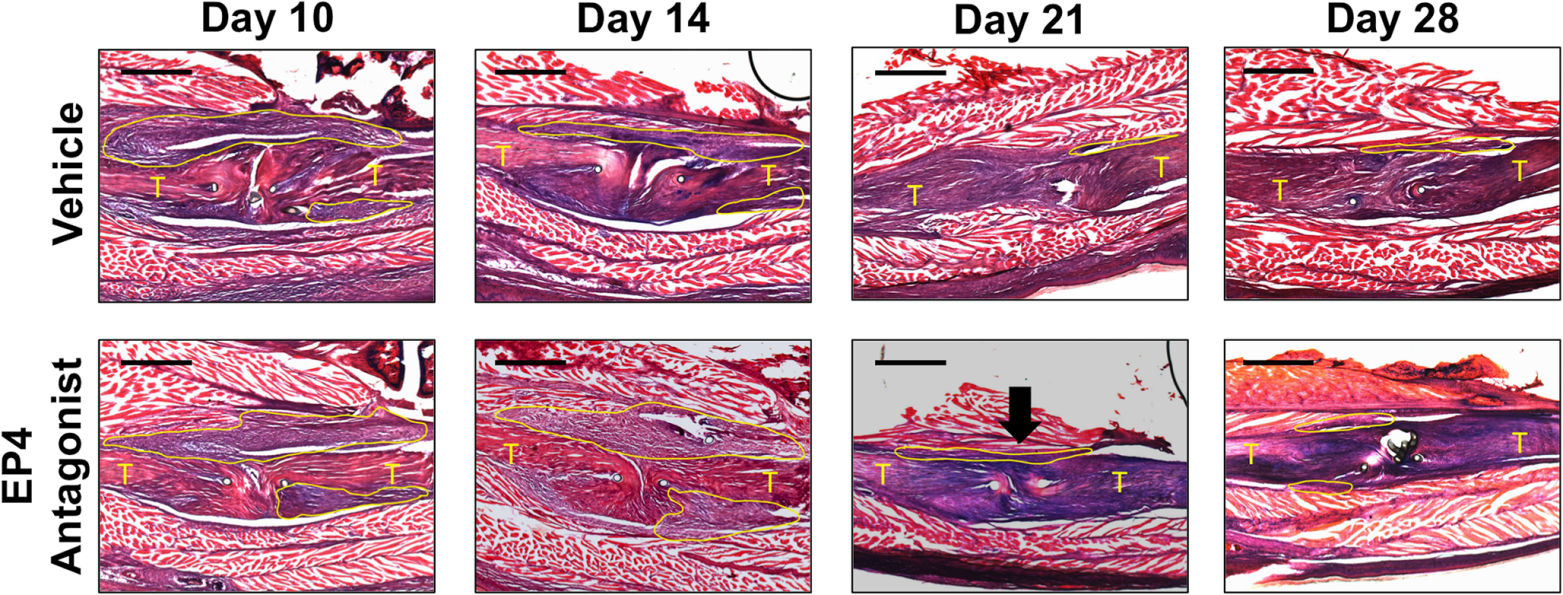
EP4 Antagonism Increased Early Granulation and Matrix Deposition around the Repair. Histologic analysis demonstrated increased granulation (outlined in yellow) and matrix deposition around the repair site in the earlier time-points of the EP4 antagonist treated repairs. This contrast is most evident at day 14. By 21 days post-repair, some granulation remained in the antagonist group (black arrow), while greater remodeling was seen in the vehicle group. Both groups have remodeled significantly by 28 days post-repair. (Alcian Blue/Hematoxylin and Orange G Staining. 5X magnification; Scale bar = 500 microns; “T” indicates tendon; Granulation tissue is outlined in yellow).

Histology was used to visualize changes in morphology and cellularity of the repair site over time, and as a function of EP4 receptor antagonism. At day 10 post-repair, both groups exhibited a large zone of granulation, with proliferating cells flanking the tendon (outlined in yellow) both superior and inferior to the repair site (Fig 4; “T” indicates proximal and distal ends of the FDL tendon). At 14 days, this response was exaggerated in the EP4 antagonist group, whereas the vehicle treated repairs began to transition to a remodeling phase with some resolution of the granulation zone. By 21 days, there was no longer a clear distinction between the tendon and granulation tissue in the vehicle treated repairs, indicating that the previously disorganized matrix had been substantially remodeled. In contrast, the EP4 antagonist repairs maintained a small degree of granulation tissue between the tendon and surrounding soft tissues (black arrow). Minimal areas of granulation remained at day 28, however both groups demonstrated greater organization of the repair site and more closely resembled the native tendon architecture.

**Fig 5:**
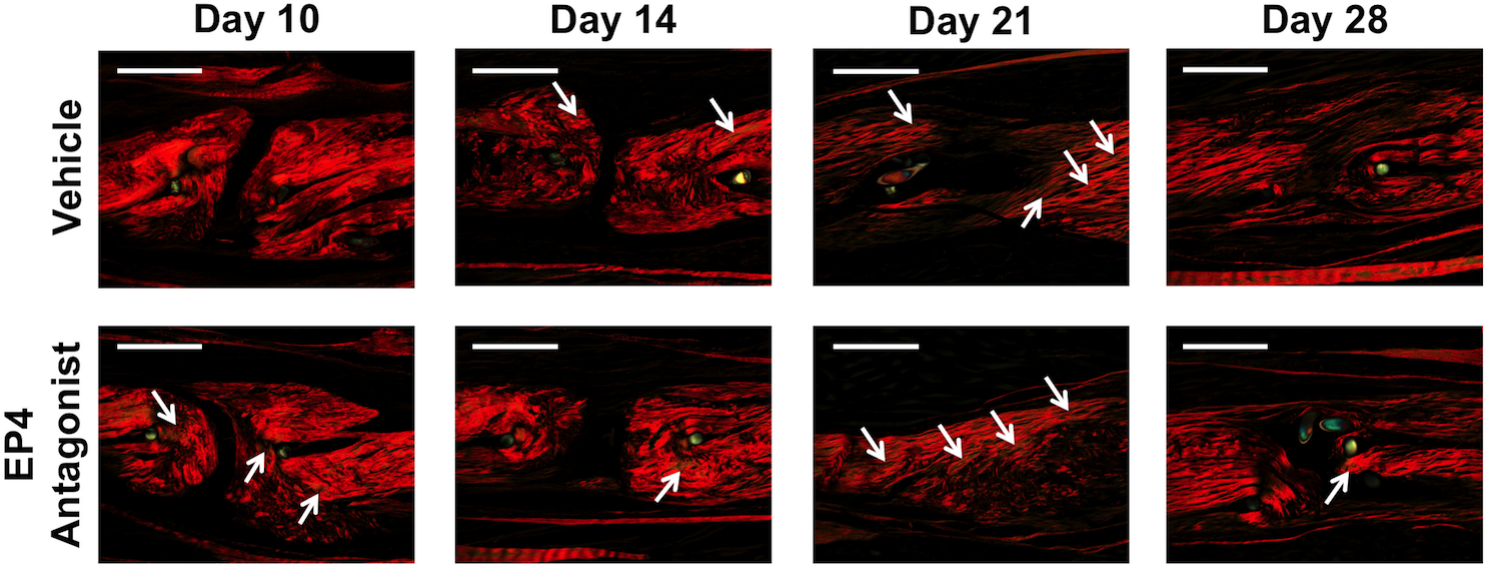
Changes in Collagen Organization by Polarized Light Microscopy. Picrosirius red staining was used to visualize changes in collagen organization over time in vehicle and EP4 antagonist treated repairs. There was increased polarization for type III collagen 10 days post-repair in the EP4 antagonist group (white arrows). Both groups had minimal organized collagen across the repair site at 10 and 14 days post-repair, though by 28 days there was evidence in both groups that the repair site was being remodeled with increases in mature collagen bridging the repair site. 10X magnification; Scale bar = 300 microns; Arrows identify areas of yellow/green staining representing Type III collagen).

Picrosirius red staining, which was used to assess changes in collagen organization, was consistent with histological observations. There was increased polarization for type III collagen at 10 days post-repair in the EP4 antagonist group (Fig 5)(white arrows), a finding that did not occur until 14 days post-repair in the vehicle group. Both groups had minimal or no evidence of organized collagen across the repair site at 10 and 14 days post-repair, though by 28 days there was evidence in both groups that the repair site was being reorganized with increases in mature Collagen I resembling the native tendon.

### EP4 Antagonism Alters Macrophage and Collagen Gene Expression during Flexor Tendon Healing

**Fig 6:**
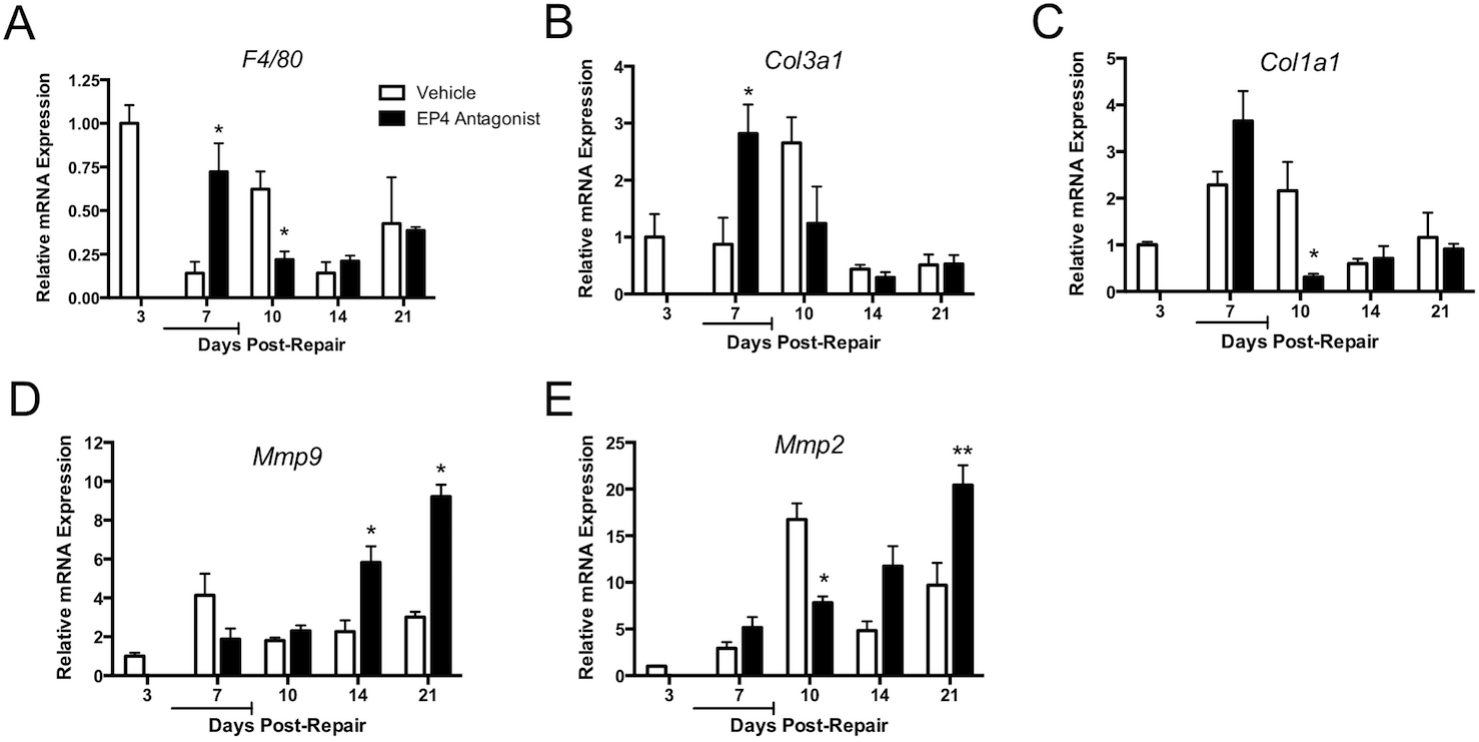
EP4 Antagonism Alters Macrophage, Collagen, and Mmp Expression After Repair. Relative mRNA expression was determined in the repair site by RT-PCR. Values were normalized to the internal control β-actin, and then normalized to expression levels at day 3 post-repair. **5A:** Expression of the macrophage marker *F4/80* is significantly increased in antagonist group (black bars) relative to vehicle at 7 days post-repair, consistent with more robust inflammation early after repair. **5B**: Significant increases in type III collagen (*Col3a1*) expression were seen in the EP4 antagonist group (black bars) at 7 days post-repair, suggesting accelerated collagen catabolism. **5C**: EP4 antagonism resulted in significant decreases in type I collagen (*Col1a1*) expression at 10 days post-repair (black bars). **5D:** *Mmp9* is associated with adhesion formation during flexor tendon repair. Significant increases were seen in the EP4 antagonist group (black bars) at 14 and 21 days post-repair, consistent with functional losses at these time-points. **5E**: *Mmp2* is associated with tissue remodeling during tendon healing. Expression of *Mmp2* increases gradually over time in the antagonist group (black bars), suggesting a response to the increased extent of granulation in these repairs. *p<0.05, **p<0.01.

The expression of genes associated with the inflammatory, proliferative and remodeling phases of healing in injured FDL tendons of EP4 antagonist and vehicle treated mice were analyzed by real-time RT-PCR and normalized to their expression level in day three repairs (Fig 6). Neither group had received treatment at this time-point; as such, day 3 has been arbitrarily labeled as vehicle at this time-point. A pan-macrophage marker, *F4/80*, was used to evaluate local changes in the macrophage response after tendon repair [36]. *F4/80* expression was highest at day 3 post-repair, and was significantly elevated in the EP4 antagonist group at day 7 relative to vehicle treated repairs at the same time point (Vehicle: 0.86-fold decrease ± 0.06; Antagonist: 0.28-fold decrease ± 0.16, p = 0.03) The differences were reversed 10 days post-repair, at which point *F4/80* expression was significantly elevated in the vehicle group relative to the antagonist group (Vehicle: 0.38-fold decrease ± 0.1; Antagonist: 0.78-fold decrease ± 0.05, p = 0.02) (Fig 6A).

Type III collagen (*Col3a1*) is associated with granulation tissue in the proliferative phase of tendon healing, and it is later remodeled to the more organized type I collagen that makes up the majority of the tendon structure during homeostasis. Both treatment groups had a transient and significant peak in *Col3a1* expression in the early stages of healing (Fig 6B). Vehicle treated repairs had a 1.7-fold increase at 10 days post-repair (Day 3: 1.0 ± 0.4; Day 10: 2.7 ± 0.5). In contrast, EP4 antagonist treated tendons had an accelerated increase in *Col3a1* expression, with a 1.8-fold increase observed 7 days post-repair (Day 3: 1.0 ± 0.4; Day 7: 2.8 ± 0.5).

PGE2 can have an inhibitory effect on the synthesis of type I collagen (*Col1a1*) [37, 38]. This association, along with the important role of type I collagen in mature tendon, led us to investigate changes in expression patterns of *Col1a1* after flexor tendon repair in the EP4 antagonist and vehicle groups (Fig 6C). At 10 days post-repair, the first time-point after discontinuing the antagonist treatment, there was a significant decrease in *Col1a1* expression in the EP4 antagonist group (Vehicle: 1.2 fold increase ± 0.6; Antagonist: 1.2-fold decrease ± 0.1, p<0.05). From day 7 to day 10, this represented a 9.7-fold decrease in the antagonist group. Neither group had increases in expression at days 14 and 21 post-repair, and expression profiles were not significantly different between the groups at time-points other than 10 days.

### Systemic EP4 Antagonist Changes the Expression of Mmp9 and Mmp2 during Repair

*Mmp9* has been implicated in scar formation during flexor tendon repair, and it is directly associated with adhesion formation [29] Consistent with the role of PGE2 in inducing expression of *Mmp9*, [39] there was decreased expression at day 7 in the EP4 antagonist group relative to vehicle, though the changes did not reach significance (Vehicle: 3.1-fold ± 1.1; Antagonist: 0.9-fold ± 0.5, p = 0.14) (Fig 6D). After discontinuing the antagonist treatment, there was a steady increase in *Mmp9* expression at days 14 (4.8-fold ± 0.8) and 21 (8.2-fold ± 0.6).

*Mmp2* is associated with the resolution of adhesions during the remodeling phase of tendon repair [30]. The vehicle treated repairs displayed a significant increase in expression at day 10 (6.8-fold ± 0.7) (Fig 6E). There was a gradual increase in *Mmp2* expression in the EP4 antagonist group from 7 to 21 days post-repair, reaching a significant 19.4-fold increase (± 2.1) at 21 days.

## Discussion

The present study demonstrates that systemic inhibition of EP4 increases matrix deposition around the repair site resulting in greater fibrous adhesion formation during FT healing. This is supported by functional data, showing impaired MTP ROM and increased gliding resistance, along with a more robust granulation response in the EP4 antagonist treated repairs. Changes in expression of *F4/80*, *Col3a1*, *Col1a1*, *Mmp9*, and *Mmp2* provide insight into the molecular events behind the phenotypic changes. The EP4 antagonist group exhibited an accelerated peak of *Col3a1* expression with decreases in *Col1a1* at earlier time-points, along with elevated expression of *F4/80* relative to vehicle. *Mmp9* expression increased at 14 and 21 days post-repair, while *Mmp2* continued to increase as time progressed, consistent with the greater extent of disorganized matrix in the antagonist group.

A major challenge in using biological approaches to improve tendon repair is achieving an optimal balance in inflammation. In the early stages after injury, the inflammatory response is essential to initiate repair and recruit cells to the site of injury. However, during the remodeling phase, excessive inflammation can have a negative effect on the healing environment and is associated with adhesions [9, 10]. Cyclooxygenase-2 (COX-2) inhibitors are common anti-inflammatory medications used to treat tendon pathology, and their use decreases levels of PGE2 [40]. The effect of Parecoxib treatment, a COX-2 inhibitor, on tendon healing has been reported, in which treatment with Parecoxib for the first 5 days after surgery significantly decreased the strength of the tendon callus, measured 8 days after surgery [13]. The detrimental effect on strength of the callus was reversed when the treatment was withheld until 6 days post-injury. These results informed our hypothesis that delayed inhibition of the prostaglandin inflammatory cascade further downstream than COX-2, at the level of PGE2-EP4 signaling, might achieve a balance between the necessary and unwanted inflammation that takes place after tendon injury. PGE2 was selected as a target for inhibition due to previous studies that have shown the negative effects of PGE2 on tendon, collagen, and their associated catabolic genes [23, 41–43]. Further, *in* vitro studies of tendon fibroblasts have shown that IL-1β treatment of tendon fibroblasts up-regulates COX-2 and stimulates EP4 receptor expression, suggesting an association with the catabolic inflammatory process in tendon pathology [23]. Measurements of cAMP in the antagonist treated repairs demonstrated that the systemic EP4 antagonist significantly decreased EP4 signaling within the repair site, and was able to modulate prostaglandin-mediated signaling pathways during FT repair in this murine model.

In this study, the strength of the repairs was no different between EP4 antagonist and vehicle treated repairs–an important finding, since a primary concern with inhibiting inflammation is that there will be a concomitant decrease in the strength of repair. The maximum load at failure slowly increased in both groups from day 10 to day 28 post-repair, which is consistent with the repair being remodeled to a more organized structure that mimics the native tendon. Along with the changes in maximum load at failure, there were no differences in the stiffness between the two groups. While maximum load at failure represents the overall strength of the repair, the stiffness is able to provide information about tissue properties other than strength that may change during the repair process. These biomechanical results were encouraging, and suggested that delayed inhibition of EP4 in our flexor tendon injury model did not detrimentally suppress the inflammatory response in terms of the biomechanical properties conferred to the healing tendon.

The functional consequence of adhesion formation is the increasing loss in digit ROM. As fibrous adhesions form between the tendon and surrounding soft tissue, there is greater resistance to the tendons gliding within the sheath. Through in situ testing, the total ROM along with the gliding resistance, a measure of the overall work of flexion [31], was assessed. The MTP ROM was significantly lower in EP4 antagonist treated repairs at 10 and 21 days post-repair, with a similar trend present at 14 days. By day 28, there were no significant differences between the two groups. Similar changes were observed in measures of gliding resistance, with significant increases in resistance in the antagonist treated repairs at 10 and 21 days post-repair. These findings suggest that there are increasing adhesions within and around the repair site in repairs treated with an EP4 antagonist, and that these adhesions are remodeled by day 28 when there are no longer differences between groups.

After tendon injury, the repair site is initially bridged by granulation tissue composed primarily of type III collagen, which is subsequently remodeled to the more organized, mature type I collagen [1]. As such, gene expression profiles of *Col1a1* and *Col3a1* are important for characterizing the catabolic and anabolic responses to tendon injury. Previous studies have shown that exogenous PGE2 can inhibit type I collagen [37, 38]. This effect is consistent with decreased *Col1a1* expression from 7 to 10 days post-repair in the antagonist group. Both treatment groups displayed a temporary peak in *Col3a1* expression, though the antagonist group had an accelerated peak in expression. Given the phenotype of robust matrix deposition with greater adhesions in the EP4 antagonist treated repairs, accelerated *Col3a1* expression suggests earlier collagen catabolism, and is consistent with early functional detriments in this group. This is consistent with changes in *F4/80* expression, a pan-macrophage marker; the antagonist group exhibits significantly higher expression at 7 days post-repair, suggesting accelerated inflammation relative to vehicle treated controls.

It has previously been shown that deletion of *Mmp9* results in reduced catabolism of native tendon with fewer adhesions after injury and repair [29]. EP4 signaling increases the expression of *Mmp9*,[39] therefore the effect of an EP4 antagonist on temporal expression of *Mmp9* was of particular interest in this study. During the period of antagonist treatment, there was an expected decrease in *Mmp9* expression relative to the vehicle control. The delayed increase in *Mmp9* is consistent with decreased ROM at 21 days post-repair in the antagonist group, since its expression stimulates tendon catabolism and increases matrix deposition around the repair. Investigations of the role of *Mmp*2 in tendon repair suggest its involvement in facilitating the transition from early granulation tissue to the more organized collagen structure [30]. The expression profile of *Mmp2* was consistent with this presumed role for the enzyme after tendon injury. Given the increased matrix deposition and granulation response in the early time-points, there is a greater extent of tissue that requires remodeling to restore the native structure of the tendon. The steady increases in *Mmp2* from 7 to 21 days post-repair suggest a response to the robust matrix deposition seen in EP4 antagonist treated repairs.

The results of this study raise important questions regarding the role of EP4 in tendon injury and repair, since the results were counter to our original hypothesis that inhibiting EP4 would attenuate adhesion formation and matrix deposition. PGE2-EP4 signaling imparts diverse changes within different tissues, and there is evidence of both pro- and anti-fibrotic effects of this signaling pathway. In their work on PGE2 in lung fibroblasts, Huang *et al*., demonstrated an anti-fibrotic role for PGE2 and found that cAMP was a downstream mediator of decreased collagen expression in lung fibroblasts [25]. This is consistent with other studies that have shown inhibitory effects of PGE2 on fibroblast proliferation and collagen synthesis [44–46]. Since the antagonist used in this study is given systemically, it exerts its inhibitory effects across tissues and cell populations both within and outside of the repair site. While EP4 may have a pro-inflammatory role within the localized tendon environment, it remains to be seen how EP4 signaling at the systemic level contributes to the repair process in flexor tendons. These findings underscore the need to better characterize the cells that are involved with tendon repair, both locally and systemically, and to delineate the different roles of EP4 signaling across diverse cell populations. While a global inhibition of EP4 shifted the healing response toward increased matrix deposition and adhesions, a more targeted approach could achieve the desirable effect that was originally sought in this study.

This study describes the biomechanical, cellular, and molecular changes that occur following systemic EP4 antagonism in a model of flexor tendon injury. However, a number of limitations must be considered. While this model is used as a translational approach to investigate zone II injuries, we do not utilize a true zone II laceration. Given the microscopic size of hind-paw tendons in the mouse, a mid-paw laceration and repair is better able to reproduce the repair procedure used in human injuries. Also regarding the injury model, our release of the proximal myotendinous junction is not consistent with clinical practice. This is used to protect the delicate repair in the mice, since they begin active movement immediately after surgery and are not immobilized or put through controlled rehabilitation in the same way as flexor tendon repairs in the clinic. Furthermore, while relative expression of the different genes is important to observe, expression alone does not represent the full picture of translation, activity, and gene metabolism.

In summary, flexor tendon repairs treated with a systemic EP4 antagonist exhibited an increased granulation response, with greater matrix deposition, impaired early ROM, and increased gliding resistance. The biomechanical properties of the repair were no different between antagonist treatment vehicle treated repairs. Adhesion formation after primary repair of flexor tendon injuries remains a common clinical problem, and biologic approaches to attenuate the inflammatory response are needed to improve outcomes. This study highlights the complex role of EP4 signaling within the inflammatory cascade, and the need for future studies to characterize the specific cell populations involved in the different phases of tendon repair.

## Supporting Information

S1 File NC3Rs ARRIVE checklist. (PDF)

S2 File Raw quantitative data. (XLSX)
